# Dermoscopy in the COVID-19 Era: Magnifying the Gap for Clinicians

**DOI:** 10.5826/dpc.1102a69

**Published:** 2021-03-08

**Authors:** Khawar Hussain, Ashfaq A. Marghoob, Neil P. Patel

**Affiliations:** 1 Department of Dermatology, Charing Cross Hospital, Imperial College Healthcare NHS Trust, London, UK; 2 Department of Dermatology, Memorial Sloan Kettering Cancer Center, New York, USA

**Keywords:** dermoscopy, COVID-19, medical education, skin cancer, teledermatology

Dermoscopy is an invaluable tool that has improved the diagnostic ability of clinicians to differentiate between benign and malignant skin lesions by up to 25% [1]. It is practiced using a handheld device and interpretation is operator-dependent, such that expertise in this technique requires many hours of training. Its greatest use is in the diagnosis of pigmented lesions, but dermoscopy also has an important role in the diagnosis of nonmelanoma skin cancers, benign lesions, inflammatory disorders, and even parasitic infections.

The advent of teledermatology, hastened by the ongoing COVID-19 pandemic, has changed the dynamic of the doctor-patient consultation and has led to extensive replacement of face-to-face consultations with virtual consultations. For teledermatological assessment of patients with suspected skin cancer, the historic practice of performing a face-to-face full skin examination has been supplanted by macroscopic and dermoscopic photographs of a specific skin lesion. Traditionally, clinicians have been able to intuitively gather important diagnostic clues from a dynamic dermoscopic examination of skin lesions and have taken this method of examination for granted. As teledermatology generates static dermoscopic images and omits these dynamic dermoscopic clues, we are only now beginning to realize what has been lost.

Such clues and techniques include the wobble sign for intradermal nevi [2], ink test for seborrheic keratosis [3] and porokeratosis [4], furrow ink test for pigmented acral lesions [5], oblique view dermoscopy for fibrillar-pattern pigmented lesions on the sole [6], and tape stripping of hypermelanotic nevi [7]. These dynamic dermoscopic tricks augment the established dynamic clinical tests practiced for various skin lesions during face-to-face consultations, including the pinch test for dermatofibroma, the scratch test for subcorneal hematoma [8], and lifting of crusts to examine hyperkeratotic lesions.

As the field of teledermoscopy evolves, it is important that dermatologists maximize the full potential of this technique, which would include embedding dynamic dermoscopic clues into standard teledermoscopy practice. In the virtual era, this could be achieved by training medical photographers to elicit these clues; for example, the furrow ink test and accompanying dermoscopic photographs could be recommended as standard practice when photographing any pigmented lesion on the palm or sole. Potential drawbacks include the additional resource required for photographer training and the extra time needed per patient.

Additionally, where a supplementary dynamic clinical test of a skin lesion is deemed essential by the teledermatologist, such as the scratch test for a subcorneal hematoma, we should not be discouraged from asking patients to return to a conventional face-to-face dermatological consultation in this virtual era, as the stakes of a missed skin cancer diagnosis, as ever, are high.

Furthermore, despite the continuing shift towards virtual work, it is essential that we continue to provide dermatology trainees with teaching in techniques that can only be performed face-to-face, such as handheld dermoscopy, dynamic assessment of skin lesions, and full skin examinations. Neglecting these skills would regrettably lead to a new generation of dermatologists ill-equipped to conduct a thorough face-to-face examination of skin lesions.
